# Genomic adjusted radiation dose stratifies radiotherapy dosing based on tumor-specific sensitivity in HPV^+^ oropharyngeal cancer

**DOI:** 10.1172/JCI198351

**Published:** 2025-10-01

**Authors:** Sandip K. Rath, David S. Yu

**Affiliations:** Department of Radiation Oncology and Winship Cancer Institute, Emory University School of Medicine, Atlanta, Georgia, USA.

## Abstract

Uniform radiation therapy (RT) de-escalation in HPV^+^ oropharyngeal squamous cell carcinoma (OPSCC) has underperformed in clinical trials, likely due to underlying genomic heterogeneity. In this issue of the *JCI*, Ho et al. evaluated genomic adjusted radiation dose (GARD), which integrates tumor gene expression with RT dose to estimate biological effect. In 191 locoregionally advanced HPV^+^ OPSCC patients treated with definitive RT with or without chemotherapy, GARD values varied widely, despite uniform dose delivery, and independently predicted overall survival. These data support a genomically informed framework specific for HPV^+^ OPSCC patients via GARD for guiding radiation dose de-escalation strategies.

## Uniform radiation therapy underperforms in lower-risk HPV^+^ patients

HPV^+^ oropharyngeal squamous cell carcinoma (OPSCC), which represents over 70 percent of OPSCC diagnosed in the US (1–3), is among the most curable head and neck cancers. Standard regimens — typically 70 Gy delivered via volumetric modulated arc therapy or intensity modulated radiation therapy (RT) — achieve three-year overall survival (OS) rates of 93% in the lowest risk groups (4) but can be associated with profound toxicities (5). The favorable prognosis associated with clinical success in HPV^+^ OPSCC has led to American Joint Committee in Cancer downstaging of OPSCC based on HPV status (6, 7), but, paradoxically, this downstaging has also introduced a therapeutic risk by leading to use of uniform RT dose de-escalation strategies guided solely by conventional clinical factors, which may inadvertently undertreat a subset of patients (8–14). The NRG-HN005 trial illustrated this challenge, as a uniform 60 Gy regimen in low-risk patients failed to meet its phase II endpoint (9), highlighting the need for biologically informed approaches that can individualize dose while preserving disease control. In their recent *JCI* study (15), Ho et al. proposed application of a biologically informed alternative: the genomic adjusted radiation dose (GARD) (16), a model that quantifies the biological effect of a given radiotherapy dose using tumor-specific radiosensitivity derived from a validated 10-gene radiosensitivity index (RSI) signature (comprising *AR*, *c-Jun*, *STAT1*, *PKC*, *RelA*, *c-ABL*, *SUMO1*, *CDK1*, *HDAC1*, *IRF1*) (17–19) embedded within the linear quadratic model: GARD = *D* × (α + β*D*), where α = −ln(RSI)/*D*, β is fixed at 0.05 Gy^2^, *D* represents the total radiation dose delivered in Gy, α represents the linear component of cell killing due to radiation and is derived from the patient’s individual radiosensitivity using the RSI, and β represents the quadratic component of cell killing, attributed to the accumulation of multiple “single hits” or sublethal damage. Thus, a uniform physical RT dose would be expected to display more effective tumor control in an individual with a higher GARD score.

## GARD operationalizes biologic heterogeneity

Clinically similar tumors can differ profoundly in their response to radiation due to variation in DNA damage repair efficacy, cell cycle checkpoint control, and tumor-immune interactions, as radiogenomic studies have shown (20). Such heterogeneity challenges the premise that a uniform radiation dose yields equivalent biological effects across patients. Quantifying this variability is essential for moving beyond one-size-fits-all radiotherapy toward strategies that match dose to the tumor’s intrinsic biology. Ho et al. expanded on the GARD framework, reaffirming the principle that physical dose does not equate to biological dose (15). In a prior study, they assessed a clinically homogeneous cohort of 191 locoregionally advanced HPV^+^ OPSCC patients from the BD2Decide trial (21), all treated with equivalent dose in 2 Gy fractions (EQD2) values of 69–71 Gy with or without chemotherapy. GARD scores in this cohort varied widely from 15.4 to 71.7 (median, 39.1; IQR, 12.6). In the present study, Ho et al. showed that these differences, which were not apparent from tumor-node-metastasis (TNM) stage or Eastern Cooperative Oncology Group (ECOG) performance score (ECOG, 0 in 83% of patients, indicating a high level of function) were similar to earlier results in multiple cancer types, where GARD, based on the 10-gene RSI (18, 19, 22), consistently outperforms physical RT dose in predicting outcomes. Ho. et al. further demonstrated that across all definitive RT patients, each unit increase in GARD was associated with improved OS (hazard ratio [HR] = 0.941, *P* = 0.041); in the standard-of-care dose subgroup, the HR was 0.920 (*P* = 0.019). Notably, Ho. et al. showed that patients with GARD ≥42 achieved a 3-year OS of 100%, compared with 90% for GARD <42 (*P* = 0.0045). Using multivariable analysis in HPV^+^ patients, including T stage (primary tumor), N stage (spread to lymph nodes), smoking, and ECOG performance, Ho et al. found that GARD remained the only statistically significant predictor of OS (HR = 0.943, *P* = 0.046). These results align with broader pan-cancer evidence in a pooled analysis across multiple cancer types (22). Similar results have been reported in breast cancer (16, 22), pancreatic cancer (16, 22, 23), and locally advanced rectal cancer, where GARD ≥17 predicted good pathological response (AUC = 0.75) and revealed that only approximately 17% of patients’ optimal biologically calculated doses matched guideline prescriptions (16, 22, 24).

A key advance of Ho et al. is the operationalization of GARD for biologically guided dose adaptation specifically for HPV^+^ OPSCC (15). In silico modeling showed that uniform de-escalation to 60 Gy — as performed in the NRG-HN005 trial — would reduce three-year OS from 94.6% to 92.7%, primarily because 42 additional patients would become biologically underdosed (GARD <42). By contrast, a selective GARD-guided strategy, i.e., reducing dose only in patients predicted to continue to have high GARD values at 60 Gy, preserved the 94.6% OS rate while enabling safe dose reductions in approximately 16% of patients. An even more personalized approach, ensuring a minimum GARD ≥ 32 for each patient, was projected to allow dose reduction in 77.7% of the cohort, while identifying the 22.3% who would require ≥60–70 Gy to achieve optimal tumor control. This model predicted that some patients could be adequately treated with as little as 30 Gy, while others might require doses beyond the conventional 70 Gy.

## Predictive performance and clinical implications

Time-dependent receiver operating characteristic analysis in the present study (15) further underscored GARD’s predictive strength: clinical variables alone yielded an AUC of 71.20 at 3 years, GARD alone achieved an AUC of 78.26, and the combination reached an AUC of 83.81 — an absolute gain of more than 12 points over clinical factors alone. However, adding previously defined gene expression clusters conferred no additional benefit. These findings reinforce that physical dose is not synonymous with biological dose and that failing to account for radiosensitivity risks suboptimal patient management.

## Limitations of the present study

An important consideration in interpreting the results of this study (15) is that the analyzed BD2Decide dataset was restricted to OS and not local recurrence or disease-free survival, which are important outcome measures in HPV^+^ OPSCC, and was conducted over a wide range of years of treatment (2008–2017), during which delivery approaches may have advanced; moreover, over 90% of the HPV^+^ OPSCC patients received concurrent chemoradiotherapy. As such, the independent predictive contribution of GARD should be used with caution in other contexts. Furthermore, immune checkpoint inhibitors (ICIs) were not included in the treatment landscape of the analyzed cohort. The exclusion of ICI-treated patients from the BD2Decide dataset represents a growing gap, as HPV^+^ OPSCC is immunologically active and increasingly managed with ICI (25). ICIs, including nivolumab and pembrolizumab, are approved in the recurrent/metastatic setting for head and neck cancers (26, 27) and are increasing being incorporated in the latest HPV^+^ OPSCC clinical trials (9). Whether GARD remains predictive in the presence of immune modulation remains unknown. In addition, radiosensitivity in HPV^+^ OPSCCs is also likely to be dependent on additional factors, including the tumor immune microenvironment, hypoxia, and epigenetic landscape. Incorporation of these factors, such as hypoxia using ^18^F-fluoromisonidazole positron emission tomography (28), or incorporation of additional genomic, transcriptomic, and immune profiling data currently being generated from HPV^+^ OPSCCs patients treated with RT by the National Cancer Institute (NCI) Radiation Oncology Biology Integration Network are likely to further improve guidance of RT dose de-escalation strategies.

## Conclusion and future directions: toward biologically precise radiation

Use of the GARD model offers a state-of-the-art paradigm for personalizing RT for patients with HPV^+^ OPSCC. Without a biologically informed approach, empirical RT dose reduction risks undertreating the subset of patients with intrinsically fewer radiosensitive tumors, eroding the gains achieved in this highly curable population. GARD’s ability to stratify radiosensitivity offers a path to maintain cure rates while reducing treatment-related morbidity — providing a model for how precision radiation could be implemented in other cancers. The bigger picture is clear: biologically adaptive dosing with further validation in a prospective randomized trial could redefine RT from a uniform protocol to a dynamic, patient-specific intervention.

## Version 1 10/01/2025

Electronic publication
